# Potential impact of rotavirus vaccination on reduction of childhood diarrheal disease in India: An analysis of National Family Health Survey-5

**DOI:** 10.1016/j.jvacx.2023.100319

**Published:** 2023-05-23

**Authors:** Pritu Dhalaria, Sanjay Kapur, Ajeet Kumar Singh, Ajay Verma, Pretty Priyadarshini, Gunjan Taneja

**Affiliations:** a Immunization Technical Support Unit, Ministry of Health & Family Welfare, Government of India, New Delhi 110070, India; b John Snow India, New Delhi 110070, India; c Banaras Hindu University, Varanasi, Uttar Pradesh 221005, India; d Bill & Melinda Gates Foundation, New Delhi 110067, India

**Keywords:** Rotavirus vaccine, Diarrhea, Prevalence, Burden, Hospitalization, Intervention

## Abstract

Rotavirus is one of the leading causes of diarrhea in infants and young children worldwide. In this study, we investigated the impact of rotavirus vaccination on the prevalence of diarrheal disease among children under five years of age in India. Research on the impact of the rotavirus vaccine on reducing diarrheal disease is therefore important in contributing to the growing body of evidence on the effectiveness of this intervention in improving child health outcomes. We adopted multivariate logistic regression and propensity score matching analysis to examine the association between diarrhea and the rotavirus vaccine. The bivariate analysis finding shows that the prevalence of diarrhea was remarkably higher (9.1%) among children who had not received rotavirus and the prevalence was 7.5%, 7.5%, and 7.2% among children who received one dose, two doses, and three rotavirus doses (all) respectively. The result of multivariate logistic regression shows that children who received all three doses of the rotavirus vaccine were 16% less likely to experience diarrhea compared to those who did not receive any rotavirus vaccine. Our analysis also found that the prevalence of diarrhea decreased significantly in the years following the introduction of the vaccine. The results of this study suggest that the rotavirus vaccine has a significant impact on reducing childhood diarrheal disease in India. These results have the potential to inform policy decisions and enable healthcare professionals to concert their efforts in reducing the diarrheal disease burden and its timely prevention in children. The study will also contribute to the existing literature on the impact of rotavirus vaccination in reducing the prevalence of diarrhea among children in India.

## 1 Introduction

Diarrheal disease is a global public health threat accounting for the deaths of 1 in 9 children worldwide [1]. It is one of the leading causes of morbidity and mortality in children under the age of five in developing nations, however, it is both preventable and treatable. Approximately 500,000 children under 5 years of age die annually secondary to diarrhea with rotavirus as the leading cause globally [2]. The most frequent cause of severe acute gastroenteritis (AGE)/acute watery diarrhea in young children worldwide is rotavirus. Rotavirus infection produces a wide spectrum of clinical presentations ranging from asymptomatic/subclinical infection to severe, life-threatening illness with diarrhea, vomiting, and fever. Rotavirus is primarily transmitted by fecal-oral route directly from person to person and indirectly through the consumption of virus-contaminated food and water. Rotavirus can produce severe dehydrating diarrhea, almost leading to an estimated two million hospital admissions and more than 25 million outpatient visits worldwide [3], [4]. In 2015, rotavirus infection was responsible for 29.3% of all diarrheal deaths among children under 5 years mostly concentrated in low and middle-income countries [5], [6]. Other viruses such as norovirus, enteric adenovirus, astrovirus, and enterovirus are some other viruses that cause diarrhea. Still, rotavirus is considered the major contributor to the overall prevalence of diarrhea globally, especially in developing countries.

India has a high burden of diarrheal diseases, with an estimated 10% of deaths in children under five age-group. In 2013, it was estimated that in India over 78,000 pediatric deaths per year were due to rotavirus gastroenteritis, with about 59,000 of those deaths occurring in infants under the age of two [7]. According to the Global Burden of Disease Study, diarrheal diseases are the second leading cause of death in children under five in India, accounting for over 300,000 deaths annually. Apart from deaths, rotavirus infection causes excessive loss of body fluids leading to dehydration, and malnutrition, and can have a detrimental impact on physical growth and mental development in children [8], [9]. Given the significant adverse impact of the rotavirus on child health, there is a need for effective interventions to prevent and treat rotavirus infections. The most effective public health intervention is the rotavirus vaccine, which is highly effective in reducing the incidence of rotavirus diarrhea and related hospitalizations and deaths [10].

The rotavirus vaccine is administered orally as they are intended to be swallowed and absorbed via the digestive tract unlike the majority of childhood vaccinations that are injectable. Several vaccines have been developed for the prevention of rotavirus over the years. WHO has recommended the integration of rotavirus vaccination into the national immunization programs of countries, particularly in Asia and Sub-Saharan Africa, given the higher diarrheal prevalence due to rotavirus in these regions.[11] In this context, the introduction of rotavirus vaccination in India can significantly impact on reducing the burden of diarrheal disease in young children. Studies have shown that rotavirus vaccines can reduce the incidence of rotavirus diarrhea by up to 60–70%.[12] The introduction of rotavirus vaccines in India was expected to prevent approximately 27,000 deaths, 220,000 hospitalizations, and 3.4 million outpatient visits due to diarrhea annually [13], [14]. According to the rotavirus site surveillance study introduction of rotavirus vaccination has made a significant impact on reducing the cases of rotavirus diarrheal infection and the burden has been known to decline in India [15].

Since 2015, two indigenously developed oral rotavirus vaccines (ROTAVAC by Bharat Biotech; ROTASIIL by Serum Institute of India) have been licensed by the Drug Controller General of India (DCGI), and both obtained the WHO prequalification in 2018 [16]. The introduction of the rotavirus vaccine in India’s Universal Immunization Program is a significant step in disease prevention. The Rota Virus Vaccine — an indigenous monovalent vaccine was introduced in 2016 in India’s Universal Immunization Program. The vaccine was initially introduced in four states — Andhra Pradesh, Haryana, Himachal Pradesh, and Odisha. Over the years, in a phased manner, the vaccine was gradually introduced in other states, and by 2019, it was introduced to PAN India [17]. In 2018 Rotasiil vaccine was introduced in the national immunization program. Presently, Rotasiil is being used in ten Indian states and UTs, whereas Rotavac is being utilized in twenty-six states and UTs. The introduction of rotavirus vaccination has not only led to a decline in infant death but has also reduced the cases of inpatient and outpatient care [18], [19], [20]. In many countries and regions including India, the impact and efficacy of rotavirus vaccination have been examined within surveillance networks by observing trends in rotavirus hospitalizations before and after vaccine introduction, and/or by case-control techniques to estimate vaccine effectiveness [21], [22], [23], [24], [25].

In the Indian scenario, data available to estimate the burden of rotavirus infection is largely limited to rotavirus surveillance studies based in hospital clinical settings in a controlled environment. Apart from vaccination, to reduce the burden of the virus and the resulting diarrheal cases there is a need to study follow preventive measures like safe sanitation practices and hygiene, and clean drinking water. According to surveillance data, rotavirus was found to be highly prevalent (40%) among children hospitalized with diarrhea across the country [26], [27]. Research on household-based survey data is therefore needed to understand the epidemiology of diarrhea post-vaccine introduction in India [3], [25]. This study captures the impact of rotavirus vaccination on the reduction of diarrheal disease utilizing the National Family Health Survey-5 household-based survey data.

According to the National Family Health Survey-4 (2015–16), the diarrheal prevalence was about 9.2% in children under five in the last 2 weeks preceding the survey but in NFHS-5 (2019–21) the prevalence reduced to 7.3% [28], [29]. In absolute terms, there is a 20.6% reduction in the prevalence of diarrhea between the two rounds of the survey. Research on the impact of the rotavirus vaccine on reducing diarrheal disease is therefore important in contributing to the growing body of evidence on the effectiveness of this intervention in improving child health outcomes. Therefore, the present study aims to investigate the impact of rotavirus vaccination on the reduction of diarrheal disease in a specific population, with a focus on the prevalence of diarrheal disease among vaccinated and non-vaccinated individuals in India using the most recent round of the national representative survey.

## 2 Method & materials

### 2.1 Data source

National Family Health Survey 2019–21 (NFHS-5) is the fifth round of the NFHS series since 1992–93. The objectives of NFHS-5 are to provide information on health indicators and socio-economic characteristics of women, children, and men that can help policymakers to examine the progress or status of health conditions over the years. Particularly, NFHS-5 focused on fertility, infant and child mortality, maternal and child health, and other important health and welfare indicators at national, state, and district levels in India [29].

NFHS-5 (2019–21) is a national state/union territory and district-level representative sample survey that adopted a stratified two-stage sampling design to gather information on health indicators from eligible populations/respondents. First, the sample of primary sampling units (villages in rural areas and Census Enumeration Blocks in urban areas) was selected from the sampling frame with probability proportional to size (PPS). Secondly, a random systematic sampling technique was used to select the household of eligible women (15–49 years) from the village and Census Enumeration Blocks (CEBs) in urban areas. NFHS-5 (2019–21) collected information on health indicators from 724,115 women and 101,839 men from 636,699 households and 30,198 PSUs. NFHS-2019–21 was conducted in two phases (Phase-I from 17 June 2019 to 30 January 2020 covering 17 states and 5 UTs and Phase-II from 2 January 2020 to 30 April 2021 covering 11 states and 3 UTs).

### 2.2 Explanatory variable

In NFHS-5 (2019–21) data now includes information on Rotavirus Vaccination (RVV) for the first time among children under 5 years in India, which serves as the key explanatory variable. There were two sources to collect information about the rotavirus vaccination — vaccination card and mother report/recall. In India, three doses of the rotavirus vaccine are given under the Universal Immunization Program. The first dose is given when the child is 6 weeks old, the second at 10 weeks, and the third at 14 weeks. The other explanatory variables were — age, sex, birth order of the child, mother's education level, marital status, occupation, religion, place of residence, social group (caste), place of delivery, place of vaccination, partner’s level of education, and occupation. Household wealth index — a composite measure of the socioeconomic status of the household was used for ranking the households from poorest to richest quantiles. The type of toilet facilities, household structure, and type of drinking water was also included as explanatory variables.

### 2.3 Outcome variable

**Diarrhea:** In NFHS-5 (2019–21) collected information on diarrhea in the last two weeks preceding the survey among children under five age group who had any single episode of diarrhea.

### 2.4 Statistical methods

This study displays the distribution of the doses of rotavirus vaccine among children aged 12–35 months old by socio-economic status of children and their mothers. To estimate the distribution of the doses of rotavirus vaccine with a 95% confidence interval this study used the “svyset” Stata survey design command. The confidence intervals are constructed using a logit transform so that their endpoints always lie between 0 and 1. In addition, we have shown the distribution of diarrhea among children aged 12–35 months old with doses of rotavirus vaccine and other socio-economic status of children and their mothers. To show the effect of the rotavirus vaccine with socio-economic factors on diarrhea among children aged 12–35 months old we used multivariate logistic regression. All of the analysis has been done using STATA version 16.

### 2.5 Estimation of treatment effects

The objectives of this research are also to estimate the causal impact of rotavirus on diarrheal disease among children aged 12–35 months in India. We would like to investigate to what extent the difference observed in the outcome (Diarrhea prevalence) between treated and untreated groups of children could be attributed to the rotavirus vaccine. Treatment effects measure the causal effect of a treatment on an outcome. The treatment effect is the average causal effect of a binary (0–1) variable on an outcome variable of interest. The literature suggests that for observational data non-experimental methods particularly, propensity score matching (PSM) could be used to estimate the causal impact of an intervention [30], [31], [32]. The propensity score is defined by Rosenbaum (1983) as the conditional probability of receiving a treatment given a set of pre-treatment covariates [33].

(1) p(X)≡Pr(D=1|X)=E(D|X)

where X is the multidimensional vector of observed characteristics and D = {0, 1} is the indicator (binary variable) of exposure to treatment which is indicating whether the child has received any intervention. Mostly three seminal parameters are frequently used in the social sciences study to estimate the treatment effect. The average treatment effect (ATE), the average treatment effect on the treated (ATT), and the potential-outcome means (POMs). The present study is interested in the result of the average effect of the treatment on the treated (ATT). The average treatment effect (ATE) is the mean of the difference (Y_1i_ ─ Y_0i_) and the average treatment effect on the treated (ATT) is the mean of the difference (Y_1i_ ─ Y_0i_) among the subjects that actually receive the treatment [34].

ATT is the average treatment effect among those that receive the treatment:

(2) $$\mathrm{ATT}=E(y_{1i}-y_{0i}|t=1)$$

where y_1i_ and y_0i_ are the outcomes of treated and not treated individuals and E is the expectation.

This study used user-written kmatch command to show the treatment effect of rotavirus vaccine on diarrhea disease among children 12–35 months old [35]. Kmatch command applies propensity-score matching, using kernel matching, and nearest-neighbor matching. We estimated the ATT using an Epanechnikov kernel, nearest neighbor matching, and nearest neighbor matching with bandwidth/caliper (0.005). For post-estimation evaluation of data balancing, we estimate standardized mean differences and variance before and after matching covariates using “kmatch summarize” and kernel density estimates before and after matching were generated using the “kmatch density” command. Standardized mean differences. The standardized mean difference (SMD) is the difference in the means of each covariate between treatment groups standardized by a standardization factor so that it is on the same scale for all covariates. The standardization factor is typically the standard deviation of the covariate in the treated group when targeting the ATT. Standardized mean differences close to zero indicate a good balance. The variance ratio is the ratio of the variance of a covariate in one group to that in the other. Variance ratios close to 1 indicate good balance because they imply the variances of the samples are similar [36].

## 3 Results

Table 1 the finding shows the bivariate prevalence of the rotavirus vaccine among children 12–35 months by socio-demographic characteristics in India. The overall percentage of receiving rotavirus vaccine was 3.2% of one dose of RVV, 4.1% of two doses of RVV, and 34.2% of all three doses of rotavirus vaccine, while 58.5% of children aged 12–35 months had not received any dose of rotavirus vaccine. The percentage of the full rotavirus vaccine (all 3 doses of RVV) varies by child age, birth order, mother’s education, social group, religion, wealth status, health and nutrition education, residing with a partner, place of vaccination, and household structure.

**Table 1:** Percentage of children aged 12–35 months, who received Rotavirus vaccines by Background characteristic at any time before the survey, 2019–21.

Background characteristic	No Rotavirus	Rotavirus (One dose)	Rotavirus (Two Dose)	Rotavirus (Three doses)	Weighted
**Age of child**	% [95%, C.I.]	% [95%, C.I.]	% [95%, C.I.]	% [95%, C.I.]	Sample
12–23 months	54.9 [54.2,55.7]	3.2 [2.9,3.4]	4.4 [4.2,4.7]	37.4 [36.8,38.1]	39,989
24–35 months	62.0 [61.3,62.7]	3.2 [2.9,3.4]	3.8 [3.6,4.1]	31.0[30.3,31.7]	39,900
**Sex of child**					
Boys	58.6 [57.9,59.3]	3.2 [3.0,3.5]	4.1 [3.9,4.4]	34.1 [33.4,34.8]	41,665
Girls	58.3 [57.6,59.0]	3.1 [2.9,3.4]	4.2 [3.9,4.5]	34.4 [33.7,35.1]	38,225

**Birth order**					
1	57.9 [57.1,58.7]	3.2 [2.9,3.5]	4.1 [3.8,4.4]	34.8 [34.0,35.5]	32,359
2 to 3	58.4 [57.7,59.1]	3.0 [2.8,3.3]	4.1 [3.9,4.4]	34.4 [33.7,35.1]	39,420
4 or more	60.8 [59.4,62.2]	3.6 [3.2,4.1]	4.5 [4.0,5.1]	31.1 [29.8,32.4]	8110

**Place of residence**					
Urban	59.9 [58.7,61.1]	3.0 [2.6,3.3]	4.5 [4.0,5.0]	32.6 [31.5,33.8]	22,273
Rural	57.9 [57.3,58.5]	3.2 [3.1,3.4]	4.0 [3.8,4.2]	34.8 [34.3,35.4]	57,617

**Mother's schooling**					
No schooling	61.7 [60.6,62.8]	3.1 [2.7,3.4]	4.1 [3.7,4.6]	31.1 [30.1,32.2]	14,132
<5 years complete	67.2 [65.2,69.2]	2.9 [2.3,3.7]	3.0 [2.4,3.9]	26.8 [25.0,28.7]	3526
5–9 years complete	58.4 [57.5,59.2]	3.1 [2.9,3.4]	4.0 [3.7,4.3]	34.5 [33.7,35.3]	26,220
10 years and more complete	56.4 [55.6,57.2]	3.3 [3.0,3.6]	4.4 [4.1,4.7]	36.0 [35.2,36.8]	36,013

**Social group**					
Scheduled caste	57.4 [56.2,58.5]	3.4 [3.0,3.8]	4.3 [3.9,4.7]	35.0 [33.9,36.1]	18,268
Scheduled tribe	54.8 [53.3,56.4]	3.2 [2.7,3.8]	3.2 [2.8,3.7]	38.8 [37.2,40.3]	7819
Other backward class	56.6 [55.9,57.4]	3.2 [3.0,3.5]	4.1 [3.8,4.4]	36.1 [35.3,36.8]	34,528
Others	64.2 [63.0,65.4]	2.9 [2.6,3.2]	4.5 [4.1,5.0]	28.4 [27.3,29.5]	19,275

**Religion**					
Hindu	56.7 [56.1,57.3]	3.2 [3.0,3.4]	4.2 [4.0,4.4]	35.9 [35.3,36.5]	63,802
Muslim	69.8 [68.3,71.2]	2.9 [2.5,3.3]	3.6 [3.2,4.1]	23.7 [22.4,25.1]	12,563
Christian	55.9 [52.5,59.3]	3.0 [2.0,4.4]	5.0 [3.6,6.9]	36.1 [32.9,39.3]	1630
Others	45.0 [41.6,48.5]	3.4 [2.6,4.5]	4.6 [3.5,5.9]	47.0 [43.6,50.4]	1896

**Wealth quintile**					
Poorest	63.3 [62.4,64.3]	3.1 [2.8,3.5]	3.8 [3.5,4.1]	29.7 [28.9,30.6]	17,485
Poorer	60.8 [59.8,61.8]	3.1 [2.8,3.4]	4.1 [3.7,4.5]	32.1 [31.1,33.1]	16,981
Middle	59.0 [57.9,60.1]	2.9 [2.6,3.3]	3.7 [3.4,4.1]	34.3 [33.2,35.4]	15,968
Richer	58.1 [56.8,59.3]	3.0 [2.7,3.4]	4.3 [3.8,4.9]	34.6 [33.4,35.8]	15,656
Richest	49.2 [47.8,50.7]	3.8 [3.2,4.4]	5.0 [4.4,5.6]	42.1 [40.7,43.5]	13,801

**Health and nutrition education (in PNC)**					
No	65.4 [64.5,66.2]	3.2 [2.9,3.5]	4.3 [3.9,4.6]	27.2 [26.4,28.0]	32,566
Yes	53.7 [53.0,54.4]	3.1 [2.9,3.4]	4.1 [3.8,4.3]	39.1 [38.4,39.7]	47,324

**Mother age at birth**					
15–19 years	65.9 [64.5,67.2]	2.8 [2.4,3.3]	3.5 [3.0,4.1]	27.8 [26.6,29.1]	9376
20–29 years	57.6 [57.0,58.2]	3.2 [3.0,3.4]	4.1 [3.9,4.3]	35.1 [34.6,35.7]	59,331
30 and more years	57.0 [55.6,58.4]	3.4 [2.9,3.9]	4.9 [4.3,5.5]	34.7 [33.4,36.1]	11,183

**Birth weight**					
<2.5 kg	57.6 [56.4,58.8]	3.1 [2.8,3.5]	4.3 [3.9,4.7]	35.0 [33.9,36.2]	14,259
>=2.5 kg	58.6 [58.1,59.2]	3.2 [3.0,3.4]	4.1 [3.9,4.3]	34.1 [33.5,34.6]	65,631

**Residing with husband/partner**					
Living with partner	57.8 [57.2,58.4]	3.2 [3.0,3.3]	4.2 [4.0,4.4]	34.9 [34.3,35.4]	69,579
Staying elsewhere	63.1 [61.8,64.4]	3.2 [2.8,3.6]	3.8 [3.4,4.3]	29.9 [28.7,31.2]	9566
Single mother	60.9 [56.3,65.4]	4.5 [2.5,7.8]	3.2 [1.7,5.9]	31.5 [27.5,35.7]	745

**Sanitation**					
Improved sanitation	56.7 [56.0,57.4]	3.2 [3.0,3.5]	4.2 [3.9,4.4]	35.9 [35.2,36.5]	49,583
Improved share	62.5 [60.8,64.3]	2.6 [2.2,3.2]	3.9 [3.3,4.6]	30.9 [29.3,32.5]	7283
Unimproved sanitation	60.9 [60.0,61.8]	3.2 [2.9,3.5]	4.2 [3.8,4.5]	31.7 [30.9,32.6]	23,024

**Water Source**					
Unimproved/surface water	50.3 [47.9,52.7]	3.6 [3.0,4.4]	4.2 [3.5,5.1]	41.9 [39.6,44.2]	3130
Improved water	58.8 [58.2,59.3]	3.1 [3.0,3.3]	4.1 [3.9,4.3]	33.9 [33.4,34.4]	76,760

**Total**	**58.5 [57.9,59.0]**	**3.2 [3.0,3.3]**	**4.1 [4.0,4.3]**	**34.2 [33.7,34.7]**	79,890

Table 2 displays the prevalence of diarrhea (column 2) and multivariate logistic regression on diarrhea (column 3) among children aged 12–35 months in India. The overall prevalence of diarrhea was 8.3% among children aged 12–35 months. The prevalence of diarrhea was significantly higher at 9.1% among children who had not received the rotavirus vaccine, while among those who received the full course of the rotavirus vaccine, the prevalence was 7.2%. For children who received either one or two doses of the vaccine, the prevalence was 7.5%. The prevalence of diarrhea among children aged 12–23 months was 10.0%, while the prevalence was 6.6% among children 24–25 months. The prevalence of diarrhea was found to be similar among boys (8.6%) and girls (8.1%), indicating no significant difference by sex of the child. For children who are of first birth order, the prevalence of diarrhea was 8.0% and among children, with 4 or more birth orders the prevalence of diarrhea was 9.7%. The prevalence of diarrhea among children was 6.8% in urban areas and 8.9% in rural areas. The prevalence of diarrhea was higher (10.6%) among children with mothers with less than five years of schooling. The prevalence of diarrhea was 9.3% among children from the Schedule tribe social group. For children who belonged to Hindu and Muslim religions, the prevalence of diarrhea was 8.3% and 8.7% respectively. The prevalence of diarrhea was high (10.9%) among children those from the poorest wealth quintile and among children of teenage mothers (10.4%). The prevalence of diarrhea was 8.0% among children whose mothers received health and nutrition education and 8.6% among children who were in the low-birth-weight category (<2.5 kg). The prevalence of diarrhea was high (10.2%) among children whose fathers are not residing with family. The incidence of diarrhea in the recent two weeks was high at 10.1% among children who practiced improved sanitation and 9.3% among children from households who used unimproved water sources.

**Table 2:** Distribution of diarrhea prevalence and multivariate logistic regression of vaccinated and unvaccinated children aged 12–35 months, by background characteristics, 2019–21.

**Background characteristic**	Prevalence of Diarrhea	Logistic regression		Weighted
Number of Rotavirus	Yes % (95%, C.I.)	Odds ratio	[95%, C.I.]	Sample
No Rotavirus Vaccine	9.1 [8.7,9.5]	1	[1.00,1.00]	46,703
One dose of Rotavirus Vaccine	7.5 [6.3,8.9]	0.92	[0.80,1.07]	2530
Two doses of Rotavirus Vaccine	7.5 [6.5,8.7]	0.85*	[0.74,0.98]	3314
Three doses of Rotavirus Vaccine	7.2 [6.9,7.6]	0.84***	[0.79,0.89]	27,343

**Age of child**				
12–23 months	10.0 [9.6,10.4]	1	[1.00,1.00]	39,989
24–35 months	6.6 [6.3,7.0]	0.64***	[0.60,0.67]	39,900

**Sex of child**				
Boys	8.6 [8.2,9.0]	1	[1.00,1.00]	41,665
Girls	8.1 [7.7,8.4]	0.93**	[0.89,0.98]	38,225

**Birth order**				
1	8.0 [7.6,8.5]	1	[1.00,1.00]	32,359
2 to 3	8.3 [7.9,8.7]	1.04	[0.98,1.11]	39,420
4 or more	9.7 [8.9,10.6]	1.17**	[1.06,1.29]	8110

**Place of residence**				
Urban	6.8 [6.3,7.4]	1	[1.00,1.00]	22,273
Rural	8.9 [8.6,9.2]	0.99	[0.91,1.06]	57,617

**Mother's schooling**				
No schooling	9.8 [9.1,10.5]	1	[1.00,1.00]	14,131
<5 years complete	10.6 [9.3,12.2]	1.24***	[1.10,1.40]	3526
5–9 years complete	9.0 [8.6,9.5]	1.08	[1.00,1.16]	26,220
10 years and more complete	7.0 [6.6,7.4]	0.96	[0.88,1.04]	36,013

**Social group**				
Scheduled caste	8.9 [8.3,9.4]	1	[1.00,1.00]	18,268
Scheduled tribe	9.3 [8.4,10.1]	0.92	[0.84,1.01]	7819
Other backward class	8.0 [7.6,8.4]	0.91**	[0.85,0.97]	34,528
Others	8.0 [7.4,8.7]	0.91*	[0.83,0.99]	19,274

**Religion**				
Hindu	8.3 [8.0,8.6]	1	[1.00,1.00]	63,802
Muslim	8.7 [8.0,9.6]	1	[0.92,1.08]	12,562
Christian	7.5 [6.0,9.4]	0.87*	[0.77,0.98]	1630
Others	7.0 [5.6,8.7]	0.91	[0.79,1.05]	1896

**Wealth quintile**				
Poorest	10.9 [10.3,11.6]	1	[1.00,1.00]	17,485
Poorer	9.0 [8.5,9.6]	0.87***	[0.81,0.94]	16,981
Middle	7.9 [7.3,8.5]	0.83***	[0.76,0.91]	15,968
Richer	7.5 [6.9,8.2]	0.85**	[0.77,0.94]	15,656
Richest	5.6 [5.0,6.2]	0.74***	[0.66,0.83]	13,801

**Health and nutrition education (in PNC)**				
No	8.8 [8.4,9.3]	1	[1.00,1.00]	32,566
Yes	8.0 [7.6,8.3]	0.90***	[0.86,0.95]	47,324

**Mother age at birth**				
15–19 years	10.4 [9.5,11.3]	1	[1.00,1.00]	9376
20–29 years	8.2 [7.9,8.5]	0.79***	[0.73,0.86]	59,331
30 and more years	7.1 [6.5,7.8]	0.69***	[0.61,0.77]	11,183

**Birth weight**				
<2.5 kg	8.6 [8.1,9.3]	1	[1.00,1.00]	14,259
>=2.5 kg	8.3 [7.9,8.6]	0.91**	[0.85,0.97]	65,631

**Residing with husband/partner**				
Living with partner	8.1 [7.8,8.4]	1	[1.00,1.00]	69,579
Staying elsewhere	10.2 [9.3,11.0]	1.11*	[1.02,1.20]	9566
Single mother	9.4 [7.1,12.3]	0.99	[0.78,1.25]	745

**Sanitation**				
Improved sanitation	7.3 [7.0,7.7]	1	[1.00,1.00]	49,583
Improved share	9.2 [8.3,10.2]	1.20***	[1.09,1.31]	7283
unimproved sanitation	10.1 [9.6,10.7]	1.17***	[1.10,1.25]	23,024

**Water Source**				
Unimproved/surface water	9.3 [8.1,10.6]	1	[1.00,1.00]	3130
Improved water	8.3 [8.0,8.6]	1.04	[0.93,1.16]	76,760

**Total**	**8.3 [8.0,8.6]**			79,890

Significance: *P < 0.05; **P < 0.01; ***P < 0.001.

A binary logistic regression was applied to know the relative effect of the rotavirus vaccine with socio-demographic variables on the prevalence of diarrhea among children aged 12–35 months (Table 2). The chance of occurrence of diarrhea was 16% less likely among those children who received all rotavirus vaccines (3 doses) as compared with children who did not receive any rotavirus vaccine (OR: 0.84; 95% CI: 0.79–0.89). The odds ratio of diarrhea was 0.64 times less likely to occur among children aged 12–23 months old as compared with children aged 24–35 months (OR: 0.64; 95% CI: 0.60–0.67). The risk of diarrhea was 0.93 times less likely to occur among boys as compared with girls (OR: 0.93; 95% CI: 0.89–0.98). The chance of diarrhea increases with an increase in the birth order. The risk of diarrhea was 1.16 times more likely to occur among children born with 4 or more birth orders as compared with first-order children (OR: 1.17; 95% CI: 1.06–1.29). Surprisingly the risk of occurrence of diarrhea was 1.24 and 1.08 times more likely among children whose mothers had<5 years of schooling (OR: 1.24; 95% CI: 1.10–1.40) and 5–9 years of education (OR: 1.08; 95% CI: 1.00–1.16) as compared with children with uneducated mothers. The probability of diarrhea was 0.91 and 0.91 times less likely to occur among children from other backward classes (OR: 0.91; 95% CI: 0.85–0.97) and other social groups (OR: 0.91; 95% CI: 0.83–0.99), respectively, compared to children from Schedule Caste (SC). The risk of diarrhea was 0.74 times less likely to occur among children in the richest quintile as compared to children from the poorest wealth quintile (OR: 0.74; 95% CI: 0.66–0.83). The chance of diarrhea was 0.90 times less likely to occur among children whose mothers’ received health and nutrition education during post-natal care (OR: 0.90; 95% CI: 0.86–0.95). The risk of diarrhea was 0.79 and 0.69 times less likely to occur among children whose mother’s age at birth was 20–29 years (OR: 0.79; 95% CI: 0.73–0.86) and 30 + years (OR: 0.69; 95% CI: 0.61–0.77), respectively, as compared to teenaged mother (<19 years). The risk of diarrhea was 0.91 times less likely to occur among children whose birth weight was more than or equal to 2.5 kg compared to low birth-weighted children (OR: 0.91; 95% CI: 0.85–0.97). The odds ratio of diarrhea was 1.11 times more likely to occur among children whose father was not living with the family as compared with the children who were living with their father and mother (OR: 1.11; 95% CI: 1.02–1.20). Chance of diarrhea was 1.20 and 1.17 times more likely to occur among children who practiced the improved but shared sanitation facility other than household members (OR: 1.20; 95% CI: 1.09–1.31) and unimproved sanitation (OR: 1.17; 95% CI: 1.10–1.25) as compared to children practicing improved sanitation.

Table 3 shows the average treatment effect on treated of the Rotavirus vaccine on diarrhea among children aged 12–35 months old through the various matching algorithms. The result of propensity-score kernel matching (row 3, Table 3) shows that children (12–35 months) who received the Rotavirus vaccine at any time had 1.7% (ATT = −0.017, 95% CI = −0.021 – −0.012), low risk of diarrhea as compared with children who did not receive Rotavirus vaccine. The result from propensity-score nearest-neighbor matching (row 7, Table 3) shows that children (12–35 months) who received the Rotavirus vaccine were 1.4% (ATT = −0.014, 95% CI = −0.019 – −0.012) less risk of diarrhea as compared with children who did not receive Rotavirus vaccine. Similar treatment effects show nearest-neighbor matching but only consider a pair of observations a match if the absolute difference in the propensity score is<0.005 (half a percentage point). The average treatment effect on the treated of Rotavirus vaccine (row 11, Table 3) reduced 1.4% the risk of diarrhea among children 12–25 months old (ATT = −0.014, 95% CI = −0.019 – −0.012).

**Table 3:** Treatment effect of the Rota vaccine on diarrhea among children aged 12–35 months old.

Diarrhea (outcome)		Coef.	95% C.I	P value
Propensity-score kernel matching				
	ATT	−0.017	−0.021 - −0.012	0.001
Rota Vaccine received	Yes	0.073	0.07–0.076	0.001
	No	0.090	0.086–0.093	0.001

Propensity-score nearest-neighbour matching				
	ATT	−0.014	−0.019 - −0.01	0.001
Rota Vaccine received	Yes	0.073	0.07–0.076	0.001
	No	0.087	0.084–0.091	0.001

Propensity-score nearest-neighbour matching (Caliper)				
	ATT	−0.014	−0.019 - −0.01	0.001
Rota Vaccine received	Yes	0.073	0.07–0.076	0.001
	No	0.087	0.084–0.091	0.001

Note. 1. ATT estimates from PSM models using Epanechnikov kernel matching, PSM nearest neighbor matching, and PSM nearest neighbor matching with a caliper bandwidth of 0.005 and the common support condition imposed. The ATT represents the difference between the average outcome for those who received Rota various vaccine and their average outcome under the hypothetical scenario that they did not receive the Rotavirus vaccine in standard deviation units. After matching, treatment N = 34952 (666 unmatched) and control N = 44751 (1591 unused).

2. Control variables: Age of child, Sex of child, Birth order, Place of residence, Mother's schooling, Social group, Religion, Wealth quintile, Health and nutrition education (in PNC), Mother age at birth, Birth weight, Residing with husband/partner, Living with partner, Sanitation and Source of water.

Table 4 shows the result of the average treatment effect on treated (ATT) of the Rota vaccine on diarrhea among children aged 12–35 months old by subpopulation. Children aged 12–23 months who received the Rotavirus vaccine had significantly 2.3% (ATT = −0.023, 95% CI = −0.030 – −0.016) less risk of diarrhea than children who did not get the rotavirus vaccine. Children 12–35 months old with lower birth order had a 4.8% low probability of diarrhea (ATT = −0.048, 95% CI = −0.064 – −0.032). The effect size of the rotavirus for children of ill-treated mothers to reduce the risk of diarrhea was 3% (ATT = −0.03, 95% CI = −0.041 – −0.018). Outcomes show that if children from the poorest and poor households received the Rotavirus vaccine had a significantly lower chance of diarrhea. Among children, the poorest and poor households who received the rotavirus vaccine had 3.1% (ATT = −0.031, 95% CI = −0.042 – −0.02) and 2.5% (ATT = −0.025, 95% CI = −0.036 – −0.015) probability to reduce risk of diarrhea respectively than children from this household did not get rotavirus vaccine. Children who were given rotavirus from households with sharing and unimproved sanitation had a significantly lower chance of diarrhea respectively (ATT = −0.024, 95% CI = −0.042 – −0.007) and (ATT = −0.025, 95% CI = −0.035 – −0.016).

**Table 4:** The average treatment effect on treated (ATET) of Rota vaccine on diarrhea among children aged 12–35 months old by subpopulation.

Variables	ATT	[95% Conf. Interval]	p value
**Age of child**			
12–23 months	−0.023	−0.03 - −0.016	0.001
24–35 months	−0.006	−0.011–0	0.061

**Sex of child**		–	
Boys	−0.019	−0.026 - −0.013	0.001
Girls	−0.011	−0.017 - −0.005	0.001

**Birth order**		–	
1	−0.013	−0.02 - −0.006	0.001
2 to 3	−0.016	−0.022 - −0.009	0.001
4 or more	−0.048	−0.064 - −0.032	0.001

**Place of residence**		–	
Urban	−0.013	−0.022 - −0.005	0.002
Rural	−0.019	−0.025 - −0.014	0.001

**Mother's schooling**		–	
No schooling	−0.030	−0.041 - −0.018	0.001
<5 years complete	−0.011	−0.044–0.023	0.538
5–9 years complete	−0.013	−0.021 - −0.005	0.002
10 years and more complete	−0.014	−0.02 - −0.008	0.001

**Social group**		–	
Scheduled caste	−0.016	−0.026 - −0.006	0.002
Scheduled tribe	−0.008	−0.025–0.008	0.321
Other backward class	−0.017	−0.024 - −0.01	0.001
Others	−0.019	−0.029 - −0.01	0.001

**Religion**		–	
Hindu	−0.015	−0.019 - −0.01	0.001
Muslim	−0.018	−0.031 - −0.006	0.005
Christian	−0.017	−0.059–0.026	0.443
Others	−0.006	−0.037–0.025	0.695

**Wealth quintile**		–	
Poorest	−0.031	−0.042 - −0.02	0.001
Poorer	−0.025	−0.036 - −0.015	0.001
Middle	−0.007	−0.017–0.003	0.185
Richer	−0.010	−0.02–0	0.053
Richest	−0.010	−0.019 - −0.001	0.027

**Health and nutrition education (in PNC)**		–	
No	−0.019	−0.028 - −0.01	0.001
Yes	−0.016	−0.022 - −0.011	0.001

**Mother’s age at birth**		–	
15–19 years	−0.027	−0.042 - −0.012	0.001
20–29 years	−0.014	−0.019 - −0.009	0.001
30 and more years	−0.009	−0.022–0.004	0.159

**Birth weight**		–	
<2.5 kg	−0.018	−0.029 - −0.007	0.001
>=2.5 kg	−0.017	−0.021 - −0.012	0.001

**Residing with husband/partner**		–	
Living with partner	−0.015	−0.02 - −0.01	0.001
Staying elsewhere	−0.021	−0.035 - −0.006	0.006
Single mother	−0.018	−0.081–0.045	0.571

**Sanitation**		–	
Improved sanitation	−0.014	−0.019 - −0.008	0.001
Improved share	−0.024	−0.042 - −0.007	0.006
unimproved sanitation	−0.025	−0.035 - −0.016	0.001

**Water Source**		–	
Unimproved/surface water	−0.001	−0.026–0.023	0.916
Improved water	−0.015	−0.02 - −0.01	0.001

Note. 1. ATT estimates from PSM models using Epanechnikov kernel matching. The ATT represents the difference between the average outcome for those who received Rota various vaccine and their average outcome under the hypothetical scenario that they did not receive Rota various vaccine in standard deviation units.

**Fig. 1:**
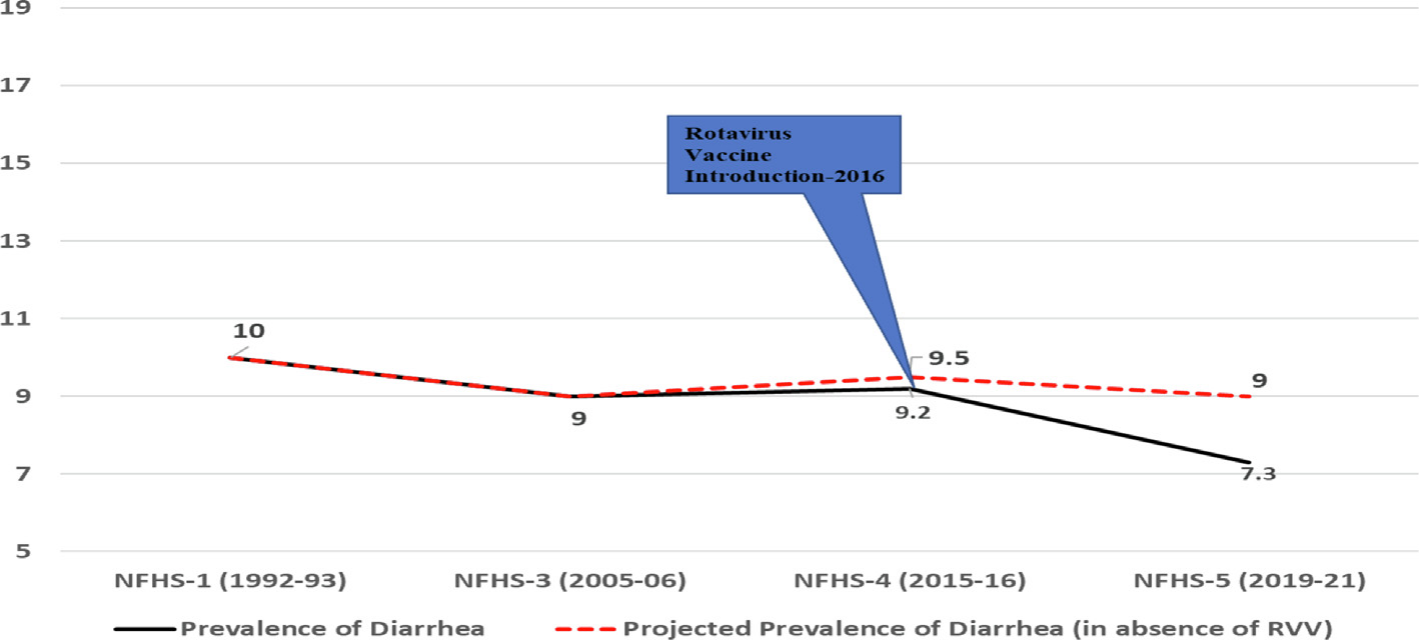
Trend of diarrheal prevalence in India.

Fig. 1 depicts the trend of diarrheal prevalence in India over the past three decades. The data indicates that during NFHS-1 (1992–93), the prevalence of diarrhea was 10%, which was reduced only by 0.8% over a span of two decades. Until NFHS-4 (2015–16), the diarrheal prevalence was 9.2%. However, after the introduction of rotavirus vaccination in India’s Universal Immunization Program in 2016, there was a significant reduction in the prevalence of diarrhea to 7.3% in NFHS-5 (2019–2021).

**Fig. 2:**
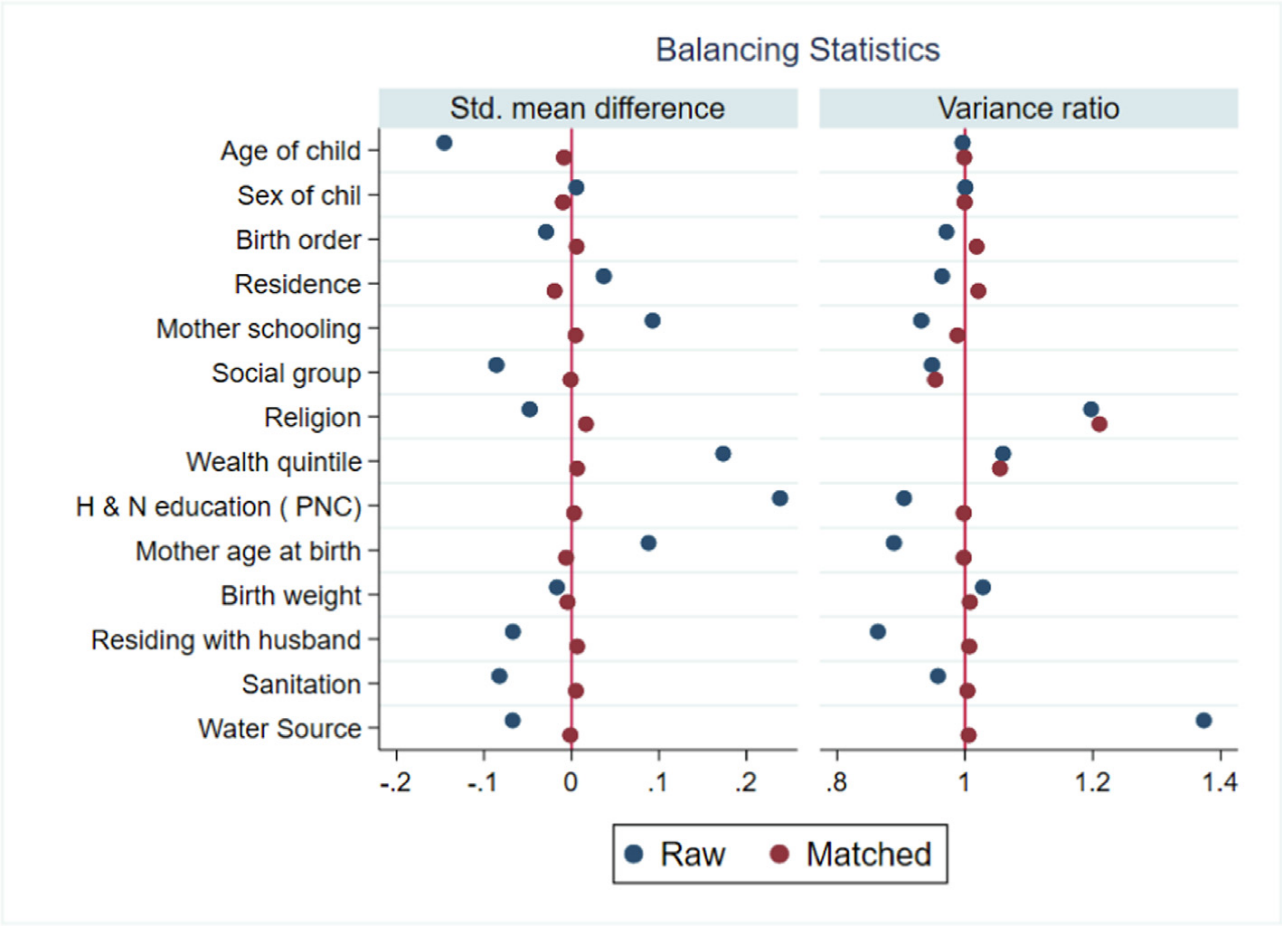
Standardized Mean difference and variance ratio of Covariates: Raw and Matched.

Fig. 2 shows the diagnostic statistics (standardized mean differences and variance ratios) that were used to evaluate covariate balance over treatment groups after the estimation of propensity score matching. The standardized differences are all close to zero and variance ratios close to 1 indicate good balance. The outcomes of the balancing statistic reveal that standardized mean difference after matching close to zero for all covariates, while variance is not equal or near about 1 of religion and wealth quintile.

**Fig. 3:**
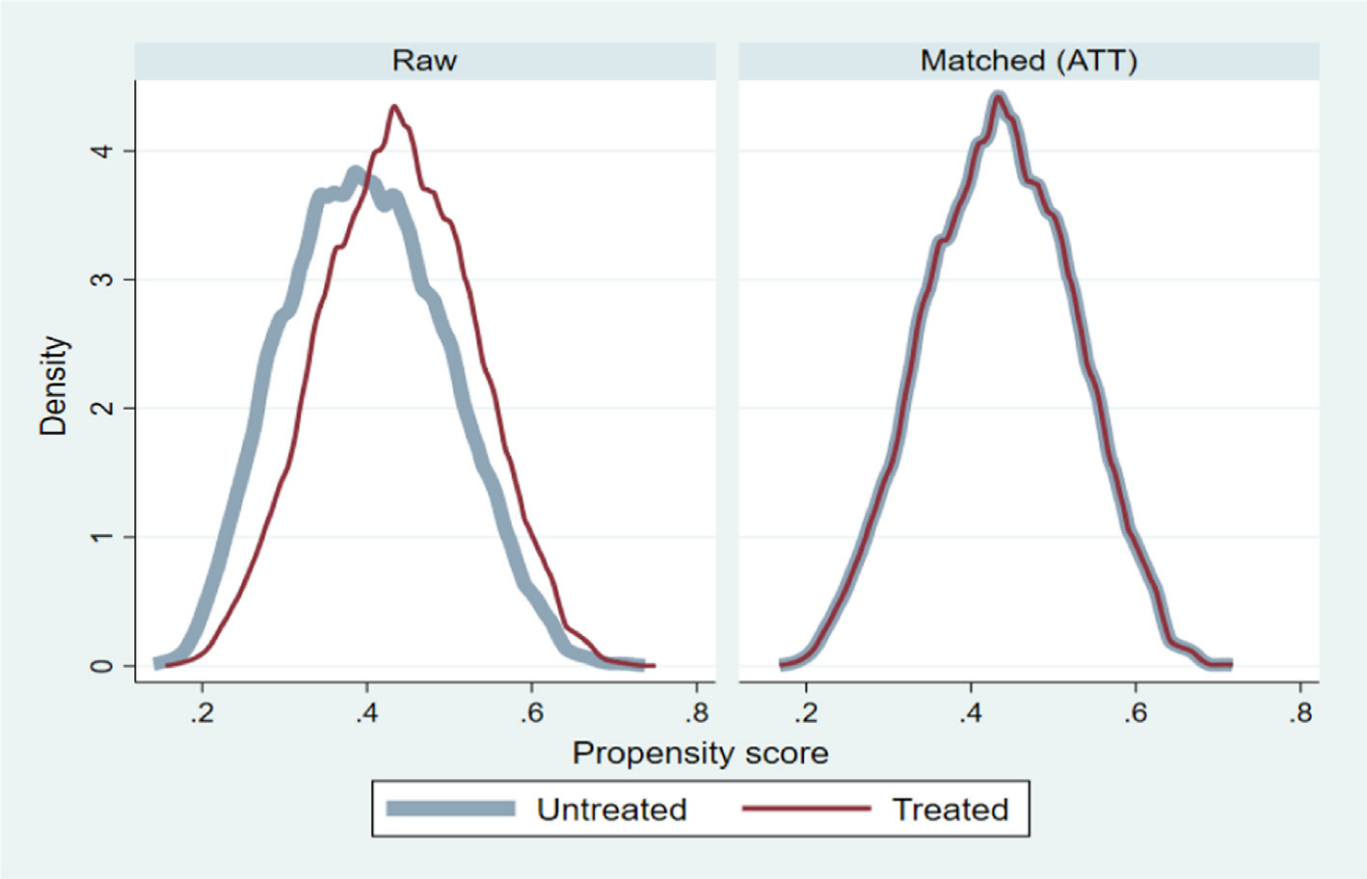
Kernel density plot of propensity score among a treated and untreated sample.

Fig. 3 shows the balance plot for the distribution of the propensity score of all covariates between treated and untreated after matching samples. To test the propensity score balance with raw and matched data, we analyzed kernel density plots of the propensity score. The output shows propensity score was not in balance in raw data for covariates, while the kernel density plots of the propensity score for the matched data do not vary over the treatment levels, so we conclude that the distribution of the propensity score of covariates was balanced.

## 4 Discussion

In developing countries, where the economic and public health burden of vaccine-preventable disease is substantial, the potential benefit of including rotavirus vaccination in national immunization programs is essential. This study aimed to identify the potential impact and contribution of Rotavirus vaccination on childhood diarrheal disease. Diarrheal disease imposes a significant economic burden on families and healthcare systems in India. Rotavirus infections have been shown to significantly affect the quality of life of children and parents during the episode, due to the illness, changes in daily activities, and other critical factors often neglected in the estimation of disease burden [37], [38]. Although rotavirus vaccination does not completely eliminate the risk of infection, breakthrough infections in vaccinated children result in a less serious illness with a shorter duration [39]. The results of this study suggest that the rotavirus vaccine has a significant impact on reducing childhood diarrheal disease in India. Our analysis found that the prevalence of diarrhea decreased significantly in the years following the introduction of the vaccine. These findings are consistent with previous studies conducted in other countries, such as Mexico and the United States, which have also shown a reduction in rotavirus infections after the introduction of the vaccine [40], [41]. Overall, the finding suggests that the rotavirus vaccine is associated with a decreased risk of diarrhea among children aged 12–35 months in India. Children who received all three doses of the rotavirus vaccine were 16% less likely to experience diarrhea compared to those who did not receive any rotavirus vaccine. Other factors associated with a decreased risk of diarrhea included being male, having a lower birth order, having a mother who is more educated, being from a higher social group or wealth quintile, having a mother who received health and nutrition education, and children whose mothers were between the ages of 20–29 at the time of the child's birth. The findings suggest that the rotavirus vaccine effectively reduces the incidence of diarrhea in children who get vaccinated and that the vaccine's effectiveness increases with the number of doses received. The finding of PSM model also suggests that the Rotavirus vaccine has a significant impact on reducing the risk of diarrhea among children aged 12–35 months in India. The propensity-score kernel matching and nearest-neighbor matching methods showed that the Rotavirus vaccine reduced the risk of diarrhea by 1.7% and 1.4%, respectively. Moreover, the average treatment effect on the treated of the Rotavirus vaccine reduced the risk of diarrhea by 1.4% among children aged 12–25 months. Several studies have evaluated the impact of rotavirus vaccination on childhood diarrheal disease in India. A systematic review and meta-analysis of six studies found that rotavirus vaccination was associated with a 41% reduction in the incidence of all-cause diarrheal disease and a 52% reduction in the incidence of rotavirus diarrhea in children under five years of age in India [3]. Another systematic review and meta-analysis of 11 studies found similar results, with a 39% reduction in the incidence of all-cause diarrheal disease and a 56% reduction in the incidence of rotavirus diarrhea in children under five years of age in India [42]. These results are important for healthcare professionals and policymakers to consider in their efforts to prevent and control diarrheal diseases in children. Moreover, preventing diarrheal disease can lead to reduced healthcare costs, leaving families with more savings to spend on education, food, and other essential needs.

Vaccine effectiveness is a measure of a vaccine’s ability to protect people against certain outcomes — infections, symptomatic illness, hospitalizations, and death [43]. In the context of this paper, it was based on the fraction of children in each age group with 0, 1, 2, or 3 doses and the expected protection of each, assuming 50% lower efficacy for a single dose in the 2-dose or 3-dose regime [44]. Along with the introduction of the rotavirus vaccine, there is a lot of improvement in safe water, sanitation, hygiene practice, awareness, and vaccine coverage which leads to a reduction in the prevalence of the disease. These findings are consistent with previous research on the effectiveness of rotavirus vaccines in preventing diarrhea [4], [6], [7]. Rotavirus infections are far more fatal in low-income nations than in high-income countries [4], [45], [46], [47]. Moreover, vaccination effectiveness is lower in low-income nations than in high-income countries [48], [49]. Unsafe drinking water, poverty, poor nutrition, inadequate sanitation, and the prevalence of illness are all known to reduce vaccination effectiveness [21]. However, in the recent decade, India has improved significantly in terms of safe drinking water (Jal Jeevan Misson), nutrition (Poshan Abhiyan), and sanitation (Swatch Bharat Misson) which played a considerable role in reducing the diarrheal prevalence in India. A systematic review and meta-analysis of randomized controlled trials found that rotavirus vaccination was associated with a 31% reduction in the risk of rotavirus diarrhea, and a 23% reduction in all-cause diarrhea [40]. Another study in Mexico found that rotavirus vaccination was associated with a 36% reduction in hospitalizations due to diarrhea [41], [50]. Earlier studies have well documented the epidemiological profile in terms of rotavirus burden in India by host characteristics like age, gender, and severity of illness as well as regional, seasonal, or genotypic variations in the circulating virus during the surveillance period [3].

The Government of India introduced the National Diarrheal Disease Control (NDDC) Program in 1978 to address the burden of diarrheal disease. In 1992–93, the program was integrated into the Child Survival and Safe Motherhood (CSSM) strategy and later incorporated into the National Rural Health Mission (NRHM) in 2005. The diarrhea control program was further strengthened by the addition of zinc (10 mg elemental zinc for 2 to 6 months infants and 20 mg/day for children greater than 6 months for 14 days) for children 3 months and above and vitamin A for all children from 9 months to 5 years of age. In 2013, the Ministry of Health and Family Welfare, Government of India (MoHFW, GoI) issued the Infant and Young Children Feeding (IYCF) guidelines bringing greater attention and commitment to promoting IYCF interventions at the health facility, community, and household levels. Since 2014, India has been implementing the Intensified Diarrhea Control Fortnight (IDCF) with the aim of achieving improved coverage of essential life-saving oral rehydration solution (ORS) and zinc dispersible tablets and increasing the practice of appropriate child feeding during diarrhea. IDCF is conducted during pre-monsoon and monsoon seasons with the aim of achieving zero child deaths due to diarrhea.

It has been more than three years since the Rotavirus vaccine was introduced nationally. It would bring a substantial impact if the vaccine is incorporated in calculating Full Immunization Coverage (FIC). This inclusion will eventually help public health officials to better monitor the effectiveness of immunization programs and ensure that children receive all vaccines under the Universal Immunization Program. Overall, including the rotavirus vaccine in full immunization coverage (FIC) calculations can help promote greater awareness of the need to encourage targeted efforts to improve coverage and protect children from rotavirus infections, and reduce diarrheal prevalence. However, introducing a new vaccine in the immunization program requires a sustained political and financial commitment. Policymakers should ensure that the necessary resources are allocated to sustain vaccine coverage in the long term, including vaccine procurement, financing, and monitoring.

It is important to note that the reduction in diarrheal disease prevalence in India would eventually have an impact on the overall prevalence of diarrheal disease globally. India has a large population with the highest birth cohort and diarrheal disease is a major health problem for children under 5 years of age group. Therefore, reducing the burden of diarrheal disease in India has the potential to significantly impact the global burden of the disease. Additionally, the success of rotavirus vaccination in India could serve as a model for other countries looking to implement similar vaccination programs.

Although the study found a reduction in diarrheal disease among those who received rotavirus vaccination, we are not in a position to determine which specific virus related to diarrhea was impacted the most. This is because the cross-sectional survey data used in the study only covered the presence of diarrheal disease in the last two weeks preceding the survey without specifying the type of virus causing diarrhea. Therefore, it is not explicit if the reduction in diarrheal disease is only due to the rotavirus covered by the vaccine or other diarrhea-causing viruses (Norovirus, Enteric Adenoviruses, etc.) not covered by the vaccine. Without this information, it is difficult to determine the extent to which the vaccine may have impacted diarrheal prevalence specific to rotavirus. This lack of specificity may limit our ability to fully attribute the observed reduction in diarrhea to the rotavirus vaccine. Therefore, while the study found a significant reduction in diarrheal disease, it may be difficult to attribute this reduction solely to the rotavirus vaccine. There is a scope for future research in exploring other factors that have an impact on diarrheal prevalence in children.

## 5 Conclusion

There are currently ongoing efforts to examine rotavirus vaccine effectiveness against diseases. Prior to vaccine development, rotavirus infection was most common in children under the age of 5. The study will contribute to the existing literature on the impact of rotavirus vaccination in reducing childhood diarrhea. It will also provide evidence for policymakers and health practitioners on the effectiveness of the rotavirus vaccine in India. In this context, the introduction of rotavirus vaccination in India has the potential to make a significant impact on reducing the burden of diarrheal disease and associated mortality in young children.

## Funding

This work was supported by the Bill & Melinda Gates Foundation through grant ID: INV-005893.

## Data availability

The data are available in the public domain and can be downloaded upon request. https://dhsprogram.com/data/available-datasets.cfm.

## Declaration of Competing Interest

The authors declare the following financial interests/personal relationships which may be considered as potential competing interests: Pritu Dhalaria reports financial support was provided by Bill & Melinda Gates Foundation.

## Data Availability

Data will be made available on request.
